# Environmental enrichment and abstinence attenuate ketamine-induced cardiac and renal toxicity

**DOI:** 10.1038/srep11611

**Published:** 2015-06-26

**Authors:** Xingxing Li, Shuangyan Li, Wenhui Zheng, Jian Pan, Kunyu Huang, Rong Chen, Tonghe Pan, Guorong Liao, Zhongming Chen, Dongsheng Zhou, Wenwen Shen, Wenhua Zhou, Yu Liu

**Affiliations:** 1 Ningbo University School of Medicine, 818 Fenghua Road, Ningbo, Zhejiang 315211, P. R. China; 2 Department of Physiology and Pharmacology, Wake Forest University School of Medicine, Medical Center Boulevard, Winston-Salem, NC 27157, USA; 3 Ningbo Kangning Hospital, 1 Zhuangyu Road, Ningbo, Zhejiang 315731, P.R.China; 4 Ningbo Addiction Research and Treatment Center, 42 Xibei Rd., Ningbo, Zhejiang 315040, P.R.China

## Abstract

The current study was designed to investigate the effect of abstinence in combination with environmental enrichment (EE) on cardiac and renal toxicity induced by 2 weeks of ketamine self-administration (SA) in rodents. In Experiment 1, one group of rats underwent ketamine SA for 14 days. In Experiment 2, the animals completed 2 weeks of ketamine SA followed by 2 and 4 weeks of abstinence. In Experiment 3, animals underwent 14 days of ketamine SA and 4 weeks of abstinence in which isolated environment (IE) and EE was introduced. The corresponding control groups were included for each experiment. Two weeks of ketamine SA caused significant increases in organ weight, Apoptosis Stimulating Fragment/Kidney Injury Molecule-1, and apoptotic level of heart and kidney. The extended length of withdrawal from ketamine SA partially reduced toxicity on the heart and kidney. Finally, introduction of EE during the period of abstinence greatly promoted the effect of abstinence on ketamine-induced cardiac and renal toxicity. The interactive effect of EE and abstinence was promising to promote the recovery of cardiac and renal toxicity of ketamine.

Ketamine is a non-competitive N-methyl-D-aspartate (NMDA) receptor antagonist which has been primarily used as a dissociative anesthetic in human and veterinary medicine^1^,^2^. In the past decade, there is a reported increase in the non-medical and unauthorized use of ketamine worldwide^3^,^4^,^5^,^6^,^7^,^8^,^9^. Ketamine use causes neurological and psychological impairment^10^,^11^,^12^. Newly accumulating data also indicate that ketamine abuse causes deleterious effects on various organs in the peripheral system^13^,^14^,^15^. For example, long-term ketamine treatment induces toxicity in the kidney, liver, bladder, and heart of mice and rabbits^16^,^17^,^18^,^19^,^20^. It has been suggestd that mitochondrial dysfunction is associated with ketamine-induced hepatotoxcity^21^,^22^,^23^,^24^. Alterations in epithelial cell-to-cell adhesion and cell coupling in the proximal kidney is hypothesized to underline ketamine-induced renal toxicity^25^. However, there is little information on therapeutic interventions of ketamine-induced functional and morphological damage in multiple organs.

Enriched environment (EE) is a combination of physical and psychological stimulation, which provides animals with multiple sensory, cognitive and motor interactions with the environment^26^. It has been shown that EE has beneficial effects on brain development, cognitive functions, and the rate of recovery from neural injury^26^,^27^,^28^,^29^,^30^,^31^. Furthermore, EE introduced during cocaine abstinence significantly reduces cue- and stress-induced cocaine self-administration in rats when compare to the non EE conditions^32^,^33^. Additionally, mice housed under the EE condition during abstinence also exhibited blunted conditioned place preference to cocaine compared with those housed under a standard condition^34^. EE is associated with a low level of stress in response to drug cues during abstinence which may attenuate drug-seeking behavior^33^,^35^,^36^. Moreover, EE also diminishes drug cue-elicited dopaminergic activity in the mesocorticolimbic circuitry during abstinence whereas isolated environment (IE) stimulates nigrostriatal dopaminergic transmission^37^. The increased cue-induced neural activity has been associated with drug relapse^38^,^39^. Thus, combination of EE and abstinence may produce a better treatment outcome for drug dependence than abstinence intervention alone.

The main purpose of this study was to determine whether EE during abstinence from ketamine treatment would attenuate chronic ketamine-induced toxicity in a rodent model of ketamine self-administration. Self-administration (SA) is one of the most relevant animal models to mimic human voluntary drug taking behavior^40^. To the best of our knowledge, the potential toxic effects on the heart and kidney exerted by ketamine have not been explored in the animal model of ketamine SA. Ketamine-induced peripheral toxicity was reported in animals non-contingently treated with ketamine for weeks or months^16^,^17^,^20^. It remains unknown as to what extent cardiac and renal damage would be produced following ketamine SA. Given the evidence of the beneficial effect of the combination of EE and abstinence on drugs of abuse in animals, it was worth determining whether abstinence in conjunction with EE would improve the recovery of cardiac and renal toxicity induced by ketamine SA.

## Results

### Cardiac and renal toxicity induced by ketamine SA

**Figure 1:**
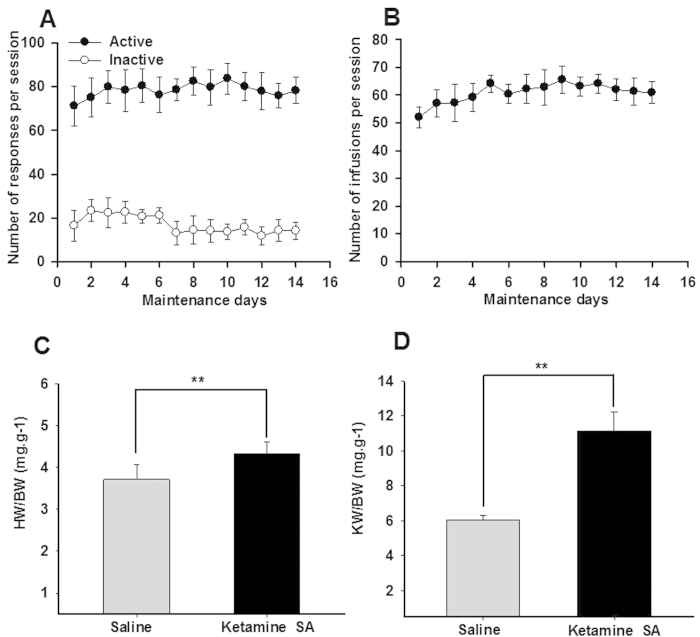
The effects of ketamine SA on hypertrophy of the rat heart and kidney. (**A**) The number of responses in the active (closed circle) and inactive (open circle) holes across 14 days of ketamine SA. (**B**) The number of infusions per session. (**C**) The heart weight and body weight ratio (HW/BW) of the control (left) and the Ketamine SA (right) groups. (**D**) The kidney weight and body weight ratio (KW/BW) of the control (left) and the Ketamine SA (right) groups. Data are expressed as mean ± SEM. * *p < 0.01 HW: heart weight; KW: kidney weight; BW: body weight.

Animals were allowed to self-administer ketamine through a choice of an active or inactive hole. Once the active hole was selected, the reinforcer (ketamine infusion) was delivered. A two-way ANOVA with repeated measurement indicated that the responses in the active hole were significantly higher than those in the inactive holes (F_(1,223)_ = 1636.917, *p* < 0.01; Fig. 1A). The number of infusions per session was stable across 14 days of ketamine SA within each group (F_(13,223)_ = 0.970, *p* = 0.670; Fig. 1B). There was no significant difference in the number of ketamine infusions between the Ketamine SA groups in different experiments.

**Figure 2:**
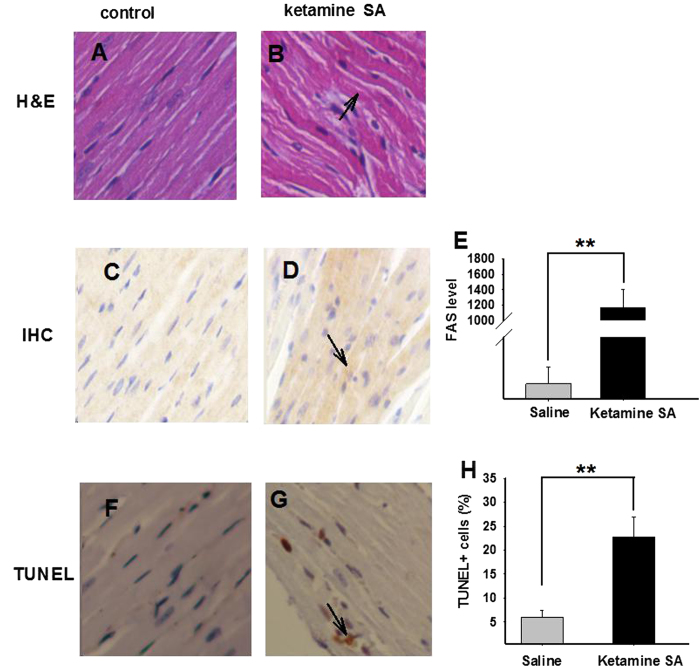
The effects of ketamine SA on morphological alterations and toxicity of the heart. (**A**,**B**) Representative images of H&E staining for a Saline and a Ketamine SA rat. Arrow represents the irregular arrangement of myocardial cells (**C**,**D**) Representative images of IHC staining for a saline and a ketamine SA rat. Arrow represents a cell with positive expression of FAS. (**E**) The level of FAS for the saline (left) and the Ketamine SA (right) groups. (**F**,**G**) Representative TUNEL staining images for a saline and a ketamine SA rat. Arrow represents an apoptotic cell. (**H**) TUNEL + cells (%) for saline (left) and ketamine SA (right) groups. Data are expressed as mean ± SEM. ** *p* < 0.01 FAS: apoptosis stimulating fragment; SA: self-administration; TUNEL: Terminal deoxynucleotidyl transferase-mediated dUTP nick end-labeling staining. The FAS level was defined as the average integral optical density.

Independent t-test revealed that both the heart weight/body ratio (HW/BW) and kidney weight/body weight ratio (KW/BW) were significantly higher in the Ketamine SA group than in the corresponding control group (HW/BW: *p* = 0.009; KW/BW: *p* < 0.01; Fig. 1C,D). Ketamine SA produced toxicity on the heart. The H&E staining results revealed that normal myocardial cells were arranged regularly (Fig. 2A). In contrast, myocardial cells were arranged irregularly and the nuclei seemed to be distorted and varied in size in the Ketamine SA group (Fig. 2B). Moreover, ketamine SA significantly increased the level of apoptosis stimulating fragment (FAS) level determined by optical density, compared with the corresponding control group (*p* < 0.01; Fig. 2C–E). Finally, TUNEL staining results indicated that the percentage of apoptotic cell number per unit area in the heart was significantly higher in the Ketamine SA group than that in the corresponding control group (*p* < 0.01; Fig. 2F–H).

**Figure 3:**
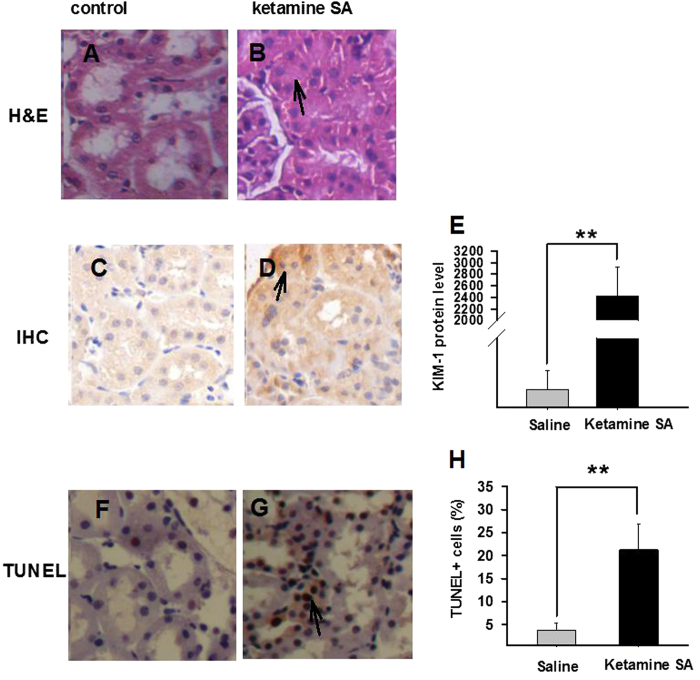
Effects of ketamine SA on morphological alterations and toxicity of the kidney. (**A**,**B**) Representative images of H&E staining for a saline and a ketamine SA rat. Arrow represents tubular atrophy. (**C**,**D**) Representative images of IHC staining for a saline and a ketamine SA rat. Arrow represents a cell with positive expression of KIM-1. (**E**) KIM-1 levels for saline (left) and ketamine SA (right) groups (**F**,**G**) Representive TUNEL staining images for a saline (left) and a ketamine SA (right) rat. (**H**) TUNEL + cells (%) for saline (left) and ketamine SA (right) groups. Arrow represents an apoptotic cell. Data are expressed as mean ± SEM. ** *p* < 0.01. SA: self-administration; KIM-1: kidney injury molecule 1; TUNEL: Terminal deoxynucleotidyl transferase-mediated dUTP nick end-labeling staining. The KIM-1 level was defined as the average integral optical density.

Ketamine SA exerted toxicity on kidney. The H&E staining results showed that the Ketamine SA group had a greater infiltration of mononuclear cells and tubular atrophy, compared with the corresponding control group (Fig. 3A,B). Moreover, tubules appeared to be distorted and blocked (Fig. 3A,B). Independent t-test revealed that the level of Kidney Injury Molecule-1 (KIM-1) was significantly increased in the Ketamine SA group, compared with the corresponding control group (*p* < 0.01; Fig. 3C–E). Lastly, ketamine SA produced a significant increase in the number of TUNEL + cells in kidney, relative to the corresponding control group (*p* < 0.01; Fig. 3F–H).

### Effect of the duration of abstinence on cardiac and renal toxicity induced by ketamine SA

To determine whether ketamine-induced toxicity was long-lasting, cardiac and renal toxicity was examined in rats with different length of abstinence (0, 2, 4 weeks) from ketamine SA. There were a total of 6 groups including no abstinence (Ketamine SA), 2 (Ketamine SA + 2 W) and 4 (Ketamine SA + 4 W) weeks of abstinence. And three additional groups were used as their corresponding controls (Saline, Saline + 2 W, Saline + 4 W). As indicated before, there was no difference in ketamine intake among the ketamine SA groups (F_(2,335)_ = 1.717, *p* = 0.199).

**Figure 4:**
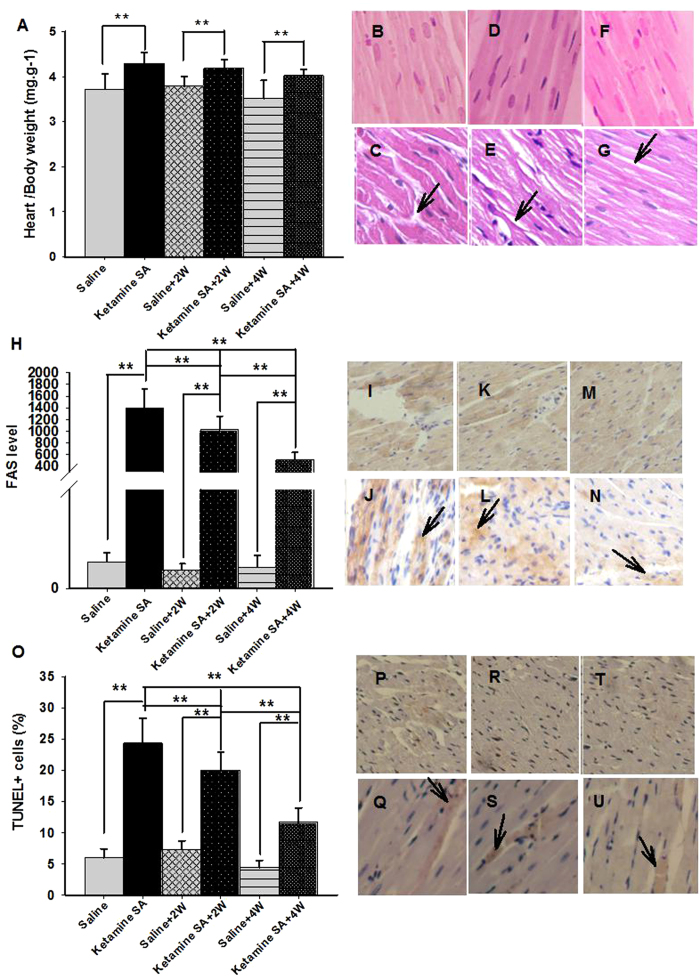
Effects of the duration of abstinence on cardiac toxicity induced by ketamine SA. (**A**) The heart and body weight ratio of Ketamine SA, Ketamine SA + 2 W and Ketamine SA + 4 W groups and their corresponding control (Saline, Saline + 2 W, Saline + 4 W) groups (**B**–**G**). Representative images of H&E staining for a Saline, a Ketamine SA, a Saline + 2 W, a Ketamine + 2 W, a Saline + 4 W and a Ketamine SA + 4 W rat. Arrow represents irregular arrangement of myocardial cell. (**H**) FAS levels of ketamine SA, ketamine SA + 2 W and ketamine SA + 4 W groups and their corresponding control (Saline, Saline + 2 W, Saline + 4 W) groups. (**I**–**N**) Representative images of IHC staining for a Saline, a Ketamine SA, a Saline + 2 W, a Ketamine + 2 W, a Saline + 4 W and a Ketamine SA + 4 W rat. Arrow represents a cell with positive expression of FAS. (**O**) TUNEL + cells (%) of the Ketamine SA, Ketamine SA + 2 W and Ketamine SA + 4 W groups and their corresponding control (Saline, Saline + 2 W, Saline + 4 W) groups (**P**–**U**) Representative images of TUNEL staining for a Saline, a Ketamine SA, a Saline + 2 W, a Ketamine + 2 W, a Saline + 4 W and a Ketamine SA + 4 W rat. Arrow represents an apoptotic cell. Data are expressed as mean ± SEM. FAS: apoptosis stimulating fragment; TUNEL: Terminal deoxynucleotidyl transferase-mediated dUTP nick end-labeling staining; SA: self-administration; W: week; HW: heart weight; BW: body weight.

A two-way ANOVA analysis revealed that there was a significant Drug (ketamine vs. saline) effect on HW/BW ratio (F_(1,41)_ = 31.855, *p* < 0.01). The effect of abstinence failed to reach significant (F_(2,41)_ = 3.081, *p* = 0.06). Post-hoc tests revealed that the HW/BW ratio in the Ketamine SA, Ketamine SA + 2 W and Ketamine SA + 4 W groups was significantly higher than that of their corresponding control groups (Ketamine SA: *p* = 0.001; Ketamine SA + 2 W: *p* = 0.015; Ketamine SA + 4 W: *p* = 0.003; Fig. 4A; Table 1). The HW/BW ratio for the Ketamine SA group was not significantly different from that for either the Ketamine SA + 2w (*p* = 0.523) or Ketamine SA + 4 W group (*p* = 0.095; Fig. 4A; Table 1). Furthermore, a two-way ANOVA revealed a significant drug and abstinence effect on the level of FAS and apoptosis (FAS/Drug: F_(1,79)_ = 287.178 *p* < 0.01; FAS/Abstinence: F_(2, 79)_ = 20.871, *p* < 0.01; Apoptosis/Drug: F_(1,55)_ = 163.830, *p* < 0.01; Apoptosis/Abstinence: F_(2,55)_ = 31.473, *p* < 0.01; Fig. 4H–U; Table 1). Post hoc tests showed that the Ketamine SA, Ketamine SA + 2 W and Ketamine SA + 4 W groups showed a significantly higher level of FAS than their corresponding control groups (Ketamine SA: *p* < 0.01; Ketamine SA + 2 W: *p* < 0.01; Ketamine SA + 4 W: *p* < 0.01; Fig. 4I–N; Table 1). The similar pattern was found for the level of apoptosis (Ketamine SA: *p* < 0.01; Ketamine SA + 2 W: *p* < 0.01; Ketamine SA + 4 W: *p* < 0.01; Fig. 4P–U; Table 1).The level of FAS was significantly lowered following 2 and 4 weeks of abstinence, relative to the Ketamine SA group (*p* < 0.01; Fig. 4J,L,N; Table 1). The similar pattern was also found for the level of apoptosis (*p* < 0.01; Fig. 4Q,S,U). Additionally, animals in the Ketamine + 4 W group showed a significantly lower level of FAS and apoptosis than those in the Ketamine + 2 W group (*p* < 0.01; Fig. 4L,N; Fig. 4S,U; Table 1).

**Figure 5:**
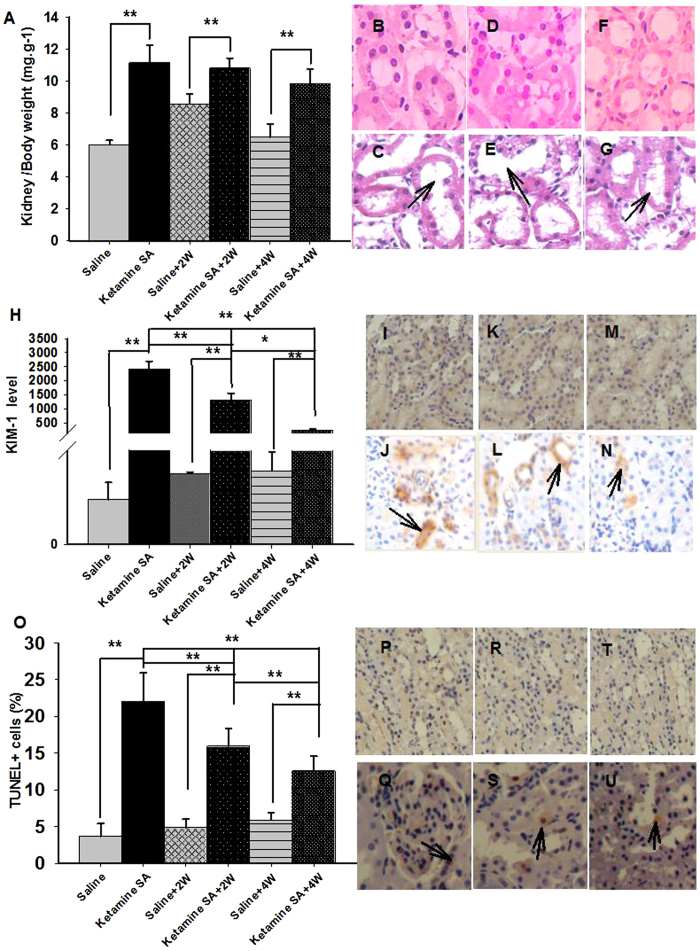
Effects of the duration of abstinence on renal toxicity induced by ketamine SA. (**A**) The kidney and body weight ratio of the Ketamine SA, Ketamine SA + 2 W and Ketamine SA + 4 W groups and their corresponding control (Saline, Saline + 2 W, Saline + 4 W) groups (**B**–**G**). Representative images of H&E staining for a Saline, a Ketamine SA, a Saline + 2 W, a Ketamine + 2 W, a Saline + 4 W and a Ketamine SA + 4 W rat. Arrow represents tubular enlargement and edema (**H**) KIM-1 levels of the Ketamine SA, Ketamine SA + 2 W and Ketamine SA + 4 W groups and their corresponding control (Saline, Saline + 2 W, Saline + 4 W) groups. (**I**–**N**) Representative images of IHC staining for a Saline, a Ketamine SA, a Saline + 2 W, a Ketamine + 2 W, a Saline + 4 W and a Ketamine SA + 4 W rat. Arrow represents a cell with positive expression of KIM-1. (**O**) TUNEL + cells (%) of the Ketamine SA, Ketamine SA + 2 W and Ketamine SA + 4 W groups and their corresponding control (**P**–**U**). Representative images of TUNEL staining for a Saline, a Ketamine SA, a Saline + 2 W, a Ketamine + 2 W, a Saline + 4 W and a Ketamine SA + 4 W rat. Arrow represents an apoptotic cell. Data are expressed as mean ± SEM. KIM-1: Kidney Injury Molecule 1; TUNEL: Terminal deoxynucleotidyl transferase-mediated dUTP nick end-labeling staining; SA: self-administration; W: week; KW: kidney weight; BW: body weight.

A two-way ANOVA indicated a significant effect of drug (saline vs. ketamine) and abstinence (0, 2, 4 weeks) on KW/BW (Drug: F_(1,41)_ = 120.423, *p* < 0.01; Abstinence: F_(1,41)_ = 7.853, *p* = 0.002; Fig. 5A; Table 1). Post-hoc tests revealed that the KW/BW ratio for the Ketamine SA, Ketamine SA + 2 W and Ketamine SA + 4 W groups was significantly higher than that for their corresponding control group (Ketamine SA: *p* < 0.01; Ketamine SA + 2 W: *p* < 0.01; Ketamine SA + 4 W: *p* < 0.01; Fig. 5A; Table 1). The KW/BW ratio of the Ketamine SA + 2 W group was not significantly different from the Ketamine SA group (*p* = 0.461), whereas the ratio for the Ketamine SA + 4 W group was significantly lower than the Ketamine SA group (*p* < 0.01; Fig. 5A; Table 1). A two-way ANOVA showed that there was a significant effect of drug and abstinence in the level of KIM-1 and apoptosis (KIM-1/Drug: F_(1,79)_ = 153.126, *p* < 0.01; KIM-1/Abstinenence: F_(2,79)_ = 34.078, *p* < 0.01; Apoptosis/Drug: F_(1,55)_ = 153.424, *p* < 0.01; Apoptosis/Abstinence: F_(2,55)_ = 11.757, *p* < 0.01; Fig. 5H,O; Table 1). The changes in KW/BW, KIM-1 and apoptosis of kidney were similar to heart. However, significant differences in the level of KIM-1 and renal cell apoptosis were evident between Ketamine SA + 2 W and Ketamine SA + 4 W group (KIM-1: *p* < 0.01; Apoptosis: *p* = 0.01; Fig. 5L,N,S,U; Table 1).

### Effects of EE abstinence on the recovery of cardiac and renal toxicity induced by ketamine SA

To determine whether EE would attenuate toxicity induced by ketamine SA, we introduced the EE and IE housing condition during the 4-weeks of abstinence following ketamine SA. Controls were saline treated animals housed in either the EE or IE condition. The number of infusions per session did not significantly differ between the EE and IE group.

**Figure 6:**
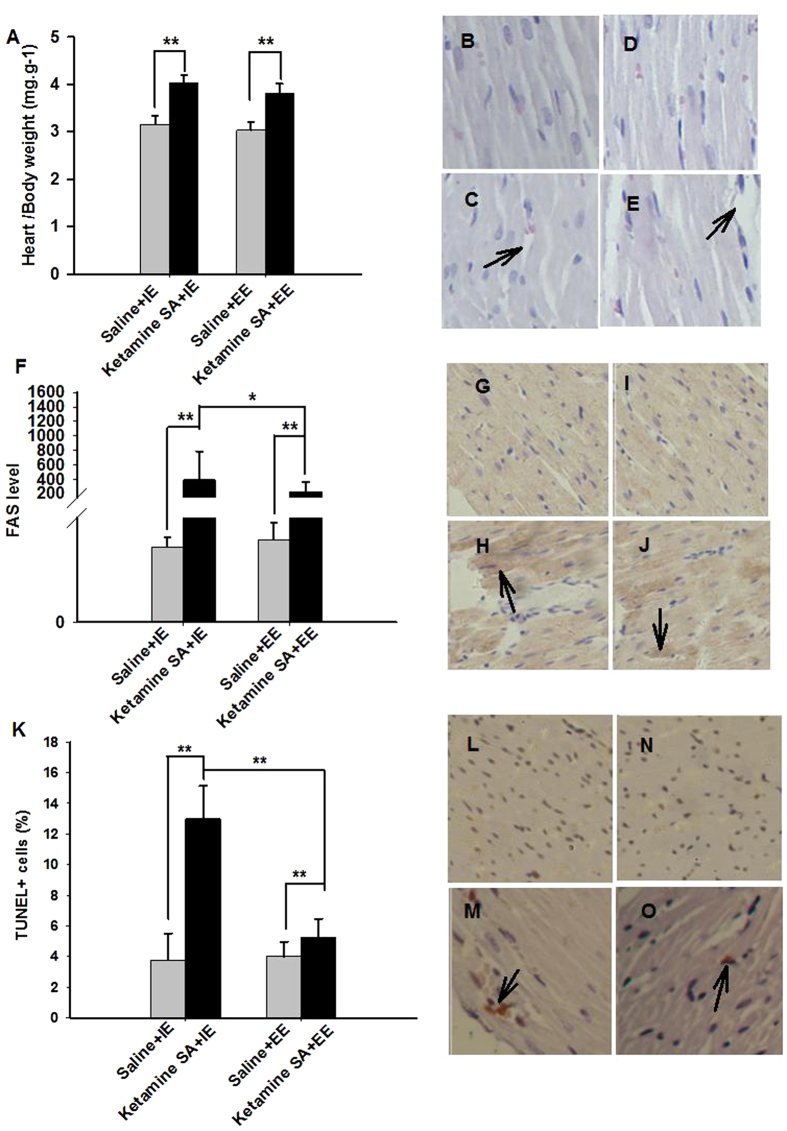
Effects of EE and IE in combination with abstinence on the recovery from ketamine-induced cardiac toxicity. (**A**) The heart and body weight ratio of the Ketamine SA + IE, Ketamine SA + EE groups and the corresponding control (Saline + IE, Saline + EE) groups (**B**–**E**). Representative images of H&E staining for a Saline + IE, a Ketamine SA + IE, a Saline + EE, and a Ketamine SA + EE rat. Arrow represents irregular arrangement of myocardial cells. (**F**) FAS levels of the Ketamine SA + IE, Ketamine SA + EE groups and the corresponding control (Saline + IE, Saline + EE) groups. (**G**–**J**). Representative images of IHC staining for a Saline + IE, a Ketamine SA + IE, a Saline + EE, and a Ketamine SA + EE rat. Arrow represents a cell with positive expression of FAS. (**K**) TUNEL + cells (%) of the Ketamine SA + IE, Ketamine SA + EE groups and the corresponding control (Saline + IE, Saline + EE) groups (**L**–**O**). Representative images of TUNEL staining for a Saline + IE, a Ketamine SA + IE, a Saline + EE, and a Ketamine SA + EE rat. Arrow represents an apoptotic cell. Data are expressed as mean ± SEM. FAS: apoptosis stimulating fragment; TUNEL: Terminal deoxynucleotidyl transferase-mediated dUTP nick end-labeling staining; IE: isolated environment; EE: enriched environment; HW: heart weight; BW: body weight.

**Figure 7:**
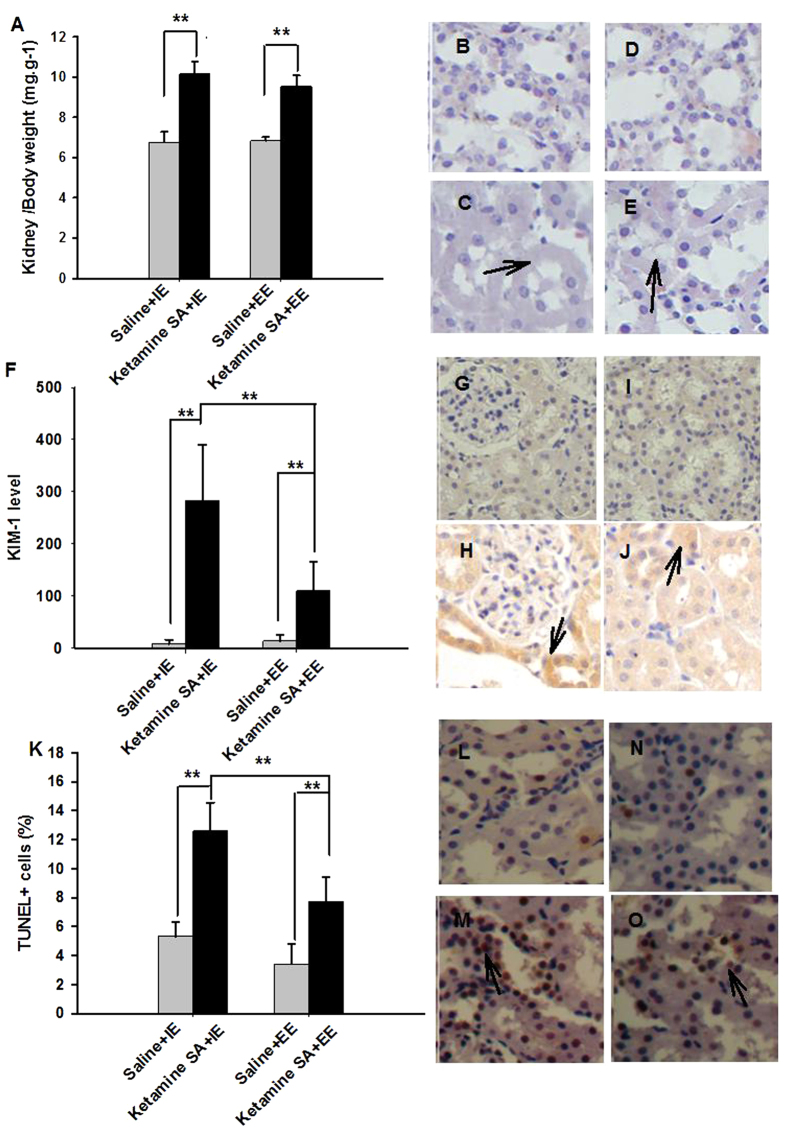
Effects of EE and IE in combination with abstinence on the recovery from ketamine-induced renal toxicity. (**A**) The kidney and body weight ratio of the Ketamine SA + IE, Ketamine SA + EE groups and the corresponding control (Saline + IE, Saline + EE) groups (**B**–**E**). Representative images of H&E staining for a Saline + IE, a Ketamine SA + IE, a Saline + EE, and a Ketamine SA + EE rat. Arrow represents tubular edema. (**F**) KIM-1 levels of Ketamine SA + IE, Ketamine SA + EE groups and the corresponding control (Saline + IE, Saline + EE) groups (**G**–**J**). Representive images of IHC staining for a Saline + IE, a Ketamine SA + IE, a Saline + EE, and a Ketamine SA + EE rat. Arrow represents a cell with positive expression of KIM-1. (**K**) KIM-1 levels of Ketamine SA + IE, Ketamine SA + EE groups and the corresponding control (Saline + IE, Saline + EE) groups. (**I**) TUNEL + cells (%) of Ketamine SA + IE, Ketamine SA + EE groups and the corresponding control (Saline + IE, Saline + EE) groups (L, M, N, O) Representive images of TUNEL staining for a Saline + IE, a Ketamine SA + IE, a Saline + EE, and a Ketamine SA + EE rat. Arrow represents an apoptotic cell. Data are expressed as mean ± SEM. KIM-1: Kidney Injury Molecule 1; TUNEL: Terminal deoxynucleotidyl transferase-mediated dUTP nick end-labeling staining; IE: isolated environment; EE: enriched environment; KW: kidney weight; BW: body weight.

A two-way ANOVA major effect of Drug (saline vs. ketamine) was found in both HW/BW (F_(1,23)_ = 123.139, *p* < 0.01) and KW/BW (F_(1,23)_ = 209.747, *p* < 0.01) ratio. The effect of Condition (IE vs. EE) was significant in the HW/BW ratio ((F_(1,23)_ = 5.471, *p* = 0.029), but not in the KW/BW ratio (F_(1,23)_ = 1.569, *p* = 0.224); Fig. 6A and 7A; Table 2). Post-hoc tests revealed that both the Ketamine SA + IE and Ketamine SA + EE group exhibited a significantly higher HW/BW ratio than their corresponding control group (Ketamine SA + IE: *p* < 0.01; Ketamine SA + EE: *p* < 0.01; Fig. 6B–E). There was also a significant difference in HW/BW and KW/BW between Ketamine SA + IE and Ketamine SA + EE group (HW/BW: *p* = 0.051; KW/BW: *p* = 0.046; Fig. 6C,E and 7C,E). For the level FAS, the was a significant effect of Drug (F_(1,51)_ = 25.437, *p* < 0.01), but not Condition (F_(1,51)_ = 2.030, *p* = 0.161). For the level of KIM-1, there was a significant effect of both Drug and Condition (Drug: F_(1,51)_ = 78.337, *p* < 0.01; Condition: F_(1,51)_ = 15.792, *p* < 0.01; Fig. 6F and 7F; Table 2). Post-hoc tests showed that the EE condition significantly decreased the level of FAS and KIM-1, compared with the IE condition (FAS: *p* = 0.045; KIM-1: *p* < 0.01; Fig. 6H,J and Fig. 7H,J). However, animals undergoing either the EE or IE abstinence still exhibited a significantly higher level of FAS and KIM-1 than their corresponding control ones (*p* < 0.01). The degree of apoptosis in the heart and kidney was also significantly different for the factor of Drug and Condition (Heart/Drug: F_(1, 47)_ = 49.202, *p* < 0.01; Heart/Condition: F_(1, 47)_ = 25.103, *p* < 0.01; Kidney/Drug: F_(1, 47)_ = 8.400, *p* = 0.006; Kidney/Condition: F_(1, 47)_ = 9.449, *p* = 0.004; Fig. 6K and 7K; Table 2). Similarly, the apoptotic level of the heart and kidney was significantly decreased by EE abstinence, relative to IE abstinence (*p* < 0.01). However, either EE or IE abstinence group exerted a significant increase in apoptosis of the heart and kidney, compared with their corresponding control group (*p* < 0.01).

## Discussion

This study was the first to investigate the effect of EE on the cardiac and renal toxicity caused by ketamine SA in rats. Two weeks of ketamine SA caused profound cardiac and renal damage, which was attenuated following 2 or 4 weeks of abstinence. Importantly, a combination of EE with abstinence produced a better effect on amelioration of ketamine-induced damages to the heart and kidney.

Our data demonstrated that ketamine SA caused dysfunction of the heart and kidney as evidenced by the significant increase in the levels of FAS and KIM-1, which is consistent with previous studies using non-contingent treatment^16^,^17^,^20^. Thus, ketamine SA may be a relevant animal of peripheral toxicity observed in human ketamine users. This model may help understand the molecular mechanisms underlying ketamine-induced toxicity. Clinical studies suggest that early intervention may produce transient and partial recovery in chronic ketamine users^14^. However, some damages can be irreversible^14^. It has not been tested whether prolonged abstinence could indeed produce greater recovery of organ injury from ketamine use. In the current study, two different periods of abstinence were used to assess the effect of abstinence on the cardiac and renal toxicity induced by ketamine SA. Both 2 and 4 weeks of abstinence lead to partial recovery from ketamine-induced toxicity with 4 weeks of abstinence showing a greater effect. This implies that some aspects of ketamine-induced peripheral toxicity may be irreversible, similar to the reports on human ketamine users^13^,^14^,^15^. One interesting finding from our study is that combination of EE and abstinence showed a greater effect on recovery from ketamine-induced peripheral toxicity. EE introduced during abstinence has been repeatedly shown to prevent cue- and stress-induced drug seeking behavior in animals, which is associated with reduced activity of brain circuitry involving in drug-induced reinstatement^32^,^37^,^41^. The duration of 4 weeks was chosen, due to the fact that 4 weeks of abstinence was not sufficient to produce a full recovery from ketamine-induced cardiac and renal toxicity. Our results showed that EE abstinence significantly reduced cardiac and renal toxicity, relative to the IE group. However, the level of FAS, KIM-1 and apoptosis in the EE abstinence group were still significantly higher than in the paired control animals. These results suggest that the combination of EE and abstinence greatly promoted the recovery from cardiac and renal damage induced by ketamine SA, but were not sufficient to produce a full recovery. It remains unknown whether a longer duration of EE and abstinence combination would produce better treatment outcomes, even a full recovery of ketamine-induced cardiac and renal toxicity.

One potential molecular mechanism underlying the effects of EE on peripheral toxicity is the reduced oxidative stress. It has been widely studied and documented that oxidative stress plays a critical role in drug-induced apoptosis of the heart and kidney^42^,^43^,^44^. Oxidative stress is known to trigger activation of a number of signaling pathways mediated by reactive oxygen species (ROSs), which subsequently activate nuclear factor-κB (NF-κB). Activation of NF-κB results in the promotion of renal damage, including interstitial fibrosis and inflammation^44^. Moreover, altered ROS levels could directly enhance the loss of epithelial properties, which is evident in animals treated with ketamine^17^. It is likely that oxidative stress could be a major contributor to the cardiac and renal impairment associated with ketamine SA. EE has been demonstrated to lower expressions of biomarkers of oxidative damage and produce anti-oxidative effects in rats with chronic cerebral hypoperfusion^45^. Pre-clinical and clinical data suggested that cognitive and physical stimulation, which is a key component of EE, lowers peripheral oxidative stress^46^. Interestingly, combined treatment of antioxidants and behavioral enrichment also reduce the levels of oxidative damage and improve the antioxidant reserve systems in the aging canine brain^47^. Taken together, it is possible that ketamine-induced oxidative damage, indicated by cardiac and renal toxicity, can be effectively attenuated by the intervention of EE during the abstinence period.

Differences in housing conditions might have profound impact on the well-being of animals^48^,^49^. For example, EE reduces heart rate and increases activity, compared with single-housing condition^50^. IE animals gained weight more rapidly than EE ones^51^,^52^,^53^. Furthermore, rats singly housed in super-enriched cages show better sleep, more exploration, and greater relative weights of the thymus gland and spleen compared to single housed rats without the EE condition^49^. Rats housed in the EE condition exhibit a less body weight gain and increased physical activity^51^,^54^. Also, a larger and more complex EE condition positively correlates with higher muscle strength in rats and rabbits^54^,^55^. Cage enrichment significantly reduces the heart rate and the systolic blood pressure in male rats, but not female rats^56^,^57^. Social and physical enrichment can induce a longer duration of sleep in rats, compared with a solely social enrichment^49^. It is likely that EE can improve the general well-being of rodents and, ultimately, enhance the recovery of organ dysfunction following ketamine SA.

Some limitations of the present study should be considered. Only male rats were used in the current study. Earlier studies have shown that the effects of EE overall generally appeared to be greater for males than for females^58^. For example, females are less responsive to the positive effects of EE and more vulnerable to retinal ischemia in social isolation^59^. Since gender differences have been implicated as a major variable of the response to the effect of drugs of abuse and treatment outcomes^60^,^61^,^62^, it would be necessary to investigate the effect of EE in female rats in the future. Secondly, it would be interesting to see whether a longer duration of EE in combination with abstinence would produce the beneficial effect to a greater extent.

Ketamine SA exerted profound cardiac and renal toxicity, which can be alleviated, but not fully recovered by abstinence. Combination of EE and abstinence could greatly promote the recovery from cardiac and renal impairment. It should be noted that clinical attempts to control the toxicity of ketamine have been relatively unsuccessful and follow-up care for life may even be needed^15^,^63^. This may reflect a greater need to include supplementary intervention into abstinence, in order to achieve more favorable treatment outcomes.

## Methods

### Animals

Male Sprague-Dawley rats, weighing approximately 300–350 at the beginning of the experiment (Zhejiang Academy of Medical Sciences, Hangzhou, Zhejiang, China), were used. The animals were housed in a temperature- and humidity-controlled room with a 12 h light/dark cycle (lights off at 07:00, on at 19:00). Food and water were abundant *ad libitum*. The experimental protocol was approved by an Institutional Review Committee for the use of Animal Subjects. All experiments were conducted in accordance with the guidelines of the Institutional Laboratory Animal Care and Use of Ningbo University.

### Surgical procedures

The details of the surgical procedures were previously described in details^64^,^65^. After 7 days of acclimation, animals were anesthetized with a combination of ketamine HCl (50 mg/kg, i.m.) and xylazine (7.5 mg/kg, i.m.). Catheters were flushed daily with sterile saline containing heparin sodium (0.4%) and penicillin sodium to maintain catheter patency and prevent infection. There were a total of 98 animals used in the current studies and 50 animals completed ketamine SA. A total of 12 animals were excluded because of not acquiring ketamine SA (N = 4) or catheter failures (N = 8).

### Ketamine SA

**Figure 8:**
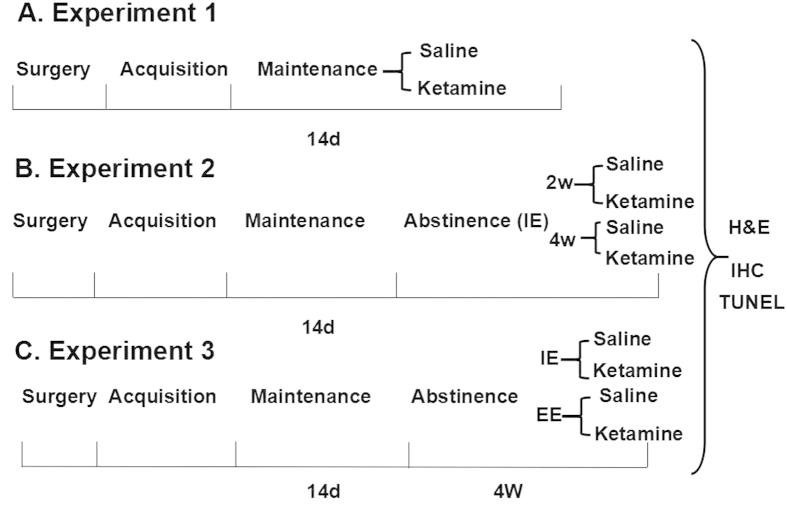
Time line for experimental design. IE: isolated environment; EE: enriched environment; W: week; D: days; H&E: Hematoxylin and Eosin Staining; IHC: Immunohistochemistry; TUNEL: Terminal deoxynucleotidyl transferase-mediated dUTP nick end-labeling staining.

Following approximately 5 days of recovery from surgery, rats were trained to SA ketamine as described previously^64^,^65^. Briefly, a nose-poke response in the left (active) hole was immediately reinforced with an injection of 0.5 mg/kg/infusion ketamine. A nose-poke response in the right (inactive) hole was considered as an inactive response which had no programmed consequences. During the acquisition phase, the animals were allowed to self-administer ketamine (0.5 mg/kg/infusion) daily on a Fixed Ratio 1 (FR1) schedule for 4 hrs. After rats acquired stable ketamine SA behavior, animals were allowed 2 hrs daily to self-administer ketamine for 14 days. The experimental procedures are summarized in Fig. 8A.

### Abstinence and housing environment

All animals were individually housed following surgery and throughout ketamine SA and saline infusion sessions. During abstinence (0, 2, or 4 weeks), rats in the IE group remained individually housed in their home cages (460 mm × 315 mm × 200 mm). IE rats were only handled briefly for scheduled bedding changes and had no visual contact with other rats housed in adjacent cages. Compared to IE, the EE group was housed together in a large plastic cage (930 mm × 615 mm × 245 mm) that contained four rats and contained bedding, two PVC pipes, two running wheels, building blocks and a number of small plastic toys. Each week, the toys were thoroughly washed and changed to maintain novelty. Saline control animals were also housed in either IE or EE condition for the same length of time as the ketamine SA groups. A cap was used to cover the catheters during the abstinence. And, catheters were flushed with heparin and saline with penisinin when necessary.

### Tissue preparation and histological study

Hematoxylin and eosin (H&E) staining was carried out to determine morphological alterations in the heart and kidney. All rats were perfused with saline and paraformaldehyde through the heart within 24 hrs of fixation after the completion of the behavioral experiment. The heart and kidneys were removed and cut into an appropriate size for the tissue specimens, and then placed in 4% paraformaldehyde to be fixed for 24 hrs. Subsequently, the specimens were placed in graded ethanol for dehydration, embedded in paraffin and cut at a thickness of 4–6 μm. The sections were deparaffinized, rehydrated and immersed in Mayer’s hematoxylin for 5–10 min. After being washed with deionized water, the sections were dipped in 0.1% acid water, immersed in 1% eosin for 5 min to develop the red color, and washed with deionized water. Finally, the sections were run through an ascending alcohol series, cleared in xylene and mounted in Permount.

### Immunohistochemical (IHC) study

According to the manufacturer’s instructions (Boshide, Wuhan, China), the heart and kidney sections were first dewaxed and rehydrated. The endogenous peroxidase activity was blocked with 3% hydrogen peroxidase for 10 min. Then the sections were incubated with goat serum blocking solution at room temperature for 20 min(m). The sections were then incubated with primary antibodies for Apoptosis Stimulating Fragment (FAS, Sigma, St. Louis, MO, USA) or Kidney Injury Molecule 1 (KIM-1, ABCAM, Cambridge, MA, USA), overnight at 4 °C. After being rinsed, the tissue sections were reacted with peroxidase-conjugated goat anti-rabbit IgG secondary antibodies at 37 °C for 30 min. Finally, all sections were dehydrated, cleared, mounted, and visualized with a DAB-based colorimetric method and Image Pro Plus 6.0 analysis software (Media Cybernetics Inc., MD, USA). The IHC index was defined as the average integral optical density.

### Terminal deoxynucleotidyl transferase-mediated dUTP nick end-labeling (TUNEL) staining

Apoptosis in the heart and kidney following ketamine SA was determined by TUNEL staining according to the manufacturer’s instructions (*In Situ* Cell Death Detection Kit, Roche, USA). The tissue sections of the heart and kidney were deparafinized and rehydrated. The samples were imaged using an Olympus CX31 microscope (Olympus, Tokyo, Japan) and analyzed in randomly selected five high power fields . TUNEL-positive cells showed dark buffy nuclei staining andTUNEL-negative cells had blue nuclei. The total number of TUNEL-positive cells per field was counted, using the software Image-Pro Plus 6.0 (Media Cybernetics Inc., MD, USA) and divided by the field area. The average apoptotic cell density was obtained from each experimental group.

## Experimental procedures

### Experiment 1: Cardiac and renal toxicity induced by ketamine SA

Two groups of animals were used in Experiment 1. One group of animals (Ketamine SA; N = 14) underwent ketamine SA for 14 days. The corresponding controls animals (N = 6) were also surgically prepared with jugular catheters. Each control animal received intravenous infusions of saline in the same temporal patterns as that self-administered ketamine. That is, the number of saline infusions and the duration between each saline infusion in the control group were the same as the average number and duration in ketamine SA group. The control group received the same number of training sessions as ketamine SA group, but because lever presses did not result in reinforcer delivery, they occurred only infrequently. The experimental procedures are summarized in Fig. 8A.

### Experiment 2: Effect of abstinence on cardiac and renal toxicity induced by ketamine SA

Experiment 2 was carried out in the same manner as in Experiment 1, except for the inclusion of an abstinence period following the maintenance of ketamine SA. One groups of rats underwent ketamine SA alone (Ketamine SA; N = 8/group). Additional two groups completed ketamine SA, with abstinence duration of 2 (Ketamine SA + 2 W) and 4 (Ketamine SA + 4 W) weeks (N = 8/group) respectively. Three group of animals were used as the control (N = 6/group). The control animals received intravenous saline infusions in the same pattern as those self-administering ketamine described in Experiment 1. Two groups of control animals underwent either 2 or 4 weeks of abstinence. The experimental procedures are summarized in Fig. 8B.

### Experiment 3: Effects of EE and IE abstinence on the recovery of cardiac and renal toxicity induced by ketamine SA

Four groups of animals were used in Experiment 3. Two groups of animals underwent 14 days of ketamine SA and 4 weeks of abstinence (N = 6/group). During the abstinence phase, one group was housed under the EE condition and the other was housed under the IE condition. Two group of animals were used as the control (N = 6/group). The control animals received intravenous saline infusions in the same pattern as those self-administering ketamine described in Experiment 1. During the abstinence phase, one control group was housed under the EE condition and the other control group was housed under the IE condition. The experimental procedures are summarized in Fig. 8C.

### Drugs

The stock ketamine was purchased (0.1 g/2 ml, Gutian Pharmaceutical Company, Fujian, China) and dissolved in saline.

### Statistical analysis

Independent t-tests were used to compare the effect of ketamine SA on cardiac and renal damage. A two-way ANOVA was used to test the effect of abstinence and housing conditions on cardiac and renal toxicity. A two-way ANOVA was used to examine SA performance. LSD *post-hoc* test was used when necessary. Changes were considered to be statistically significant when *p* < 0.05.

## Additional Information

**How to cite this article**: Li, X. *et al.* Environmental enrichment and abstinence attenuate ketamine-induced cardiac and renal toxicity. *Sci. Rep.* **5**, 11611; doi: 10.1038/srep11611 (2015).

## Figures and Tables

**Table 1:** Changes in organ weight, FAS, KIM-1 level and TUNEL + cells (%) in 2 W and 4 W group, relative to the Ketamine SA and control groups.

		**Organ/body weight(mg/g)**		**Biomarkers(FAS/KIM-1)**		**Apoptosis**	
		**Ketamine SA**	**Control**	**Ketamine SA**	**Control**	**Ketamine SA**	**Control**
Heart	Ketamine SA + 2 W	—(4.32/4.29)	↑(3.78/4.29)**	↓(1396.58/1024.93)**	↑(3.78/1024.93)**	↓(24.33/20)**	↑(7.33/20)**
	Ketamine SA + 4 W	—(4.32/4.02)	↑(3.13/4.02)**	↓ (1396.58/518.93)**	↑(4.27/518.93)**	↓(24.33/11.66)**	↑(4.38/11.66)**
Kidney	Ketamine SA + 2 W	—(11.15/10.82)	↑(8.91/10.82)**	↓(2398.29/1315.07)**	↑(52.55/1315.07)**	↓(22.08/16)**	↑(4.83/16)**
	Ketamine SA + 4 W	↓(11.15/9.82)**	↑(6.80/9.82)**	↓(2398.29/297.76)**	↑(54.63/297.76)**	↓(22.08/12.58)**	↑(5.88/12.58)**

W: weeks SA: self-administration; ↑: significant increase; ↓: significant decrease: - no significant changes. W; week; SA: self-administration; FAS: apoptosis stimulating fragment; KIM-1: Kidney Injury Molecule 1.

↑: Significant increase; ↓: Significant decrease; —: No significant changes; * *p* < 0.05; ** *p* < 0.01.

**Table 2:** Changes in organ weight, FAS, KIM-1 level and TUNEL + cells (%) in the Ketamine SA + IE and control group, relative to the KetamineSA + EE group.

**Organ**	**Group**	**Organ/body weight(mg/g)**	**Biomarkers(FAS/KIM-1)**	**Apoptosis**
		**Ketamine SA** **+** **EE**	**Ketamine SA** **+** **EE**	**Ketamine SA** **+** **EE**
Heart	Ketamine SA + IE	—(3.80/4.02)	↑(225.10/392.44)*	↑(5.25/13)**
	Saline + EE	↓(3.80/3.02)**	↓(225.10/17.78)**	↓(5.25/4)*
Kidney	Ketamine SA + IE	↑(9.55/10.15)*	↑(110.48/282.37)**	↑(7.75/12.58)**
	Saline + EE	↓(9.55/6.83)**	↑(110.48/13.56)**	↓(7.75/13.41)**

↑: significant increase; ↓: significant decrease: - no significant changes. IE: isolated environment; EE: enriched environment; FAS: apoptosis stimulating fragment; KIM-1: Kidney Injury Molecule 1.

↑: Significant increase; ↓: Significant decrease; —: No significant changes; * *p* < 0.05; ** *p* < 0.01.
